# NTDs V.2.0: “Blue Marble Health”—Neglected Tropical Disease Control and Elimination in a Shifting Health Policy Landscape

**DOI:** 10.1371/journal.pntd.0002570

**Published:** 2013-11-21

**Authors:** Peter J. Hotez

**Affiliations:** 1 Departments of Pediatrics and Molecular Virology and Microbiology, National School of Tropical Medicine at Baylor College of Medicine, Houston, Texas, United States of America; 2 Sabin Vaccine Institute and Texas Children's Hospital Center for Vaccine Development, Houston, Texas, United States of America; 3 James A. Baker III Institute at Rice University, Houston, Texas, United States of America; Natural History Museum, United Kingdom

## Abstract

The concept of the neglected tropical diseases (NTDs) was established in the aftermath of the Millennium Development Goals. Here, we summarize the emergence of several new post-2010 global health documents and policies, and how they may alter the way we frame the world's major NTDs since they were first highlighted. These documents include a new Global Burden of Disease 2010 Study that identifies visceral leishmaniasis and food-borne trematode infections as priority diseases beyond the seven NTDs originally targeted by preventive chemotherapy, a London Declaration for access to essential medicines, and a 2013 World Health Assembly resolution on NTDs. Additional information highlights an emerging dengue fever pandemic. New United Nations resolutions on women and the non-communicable diseases (NCDs) have not yet embraced NTDs, which may actually be the most common afflictions of girls and women and represent a stealth cause of NCDs. NTDs also have important direct and collateral effects on HIV/AIDS and malaria, and there is now a robust evidence base and rationale for incorporating NTDs into the Global Fund to Fight AIDS, Tuberculosis, and Malaria. “Blue marble health” is an added concept that recognizes a paradoxical NTD disease burden among the poor living in G20 (Group of Twenty) and other wealthy countries, requiring these nations to take greater ownership for both disease control and research and development. As we advance past the year 2015, it will be essential to incorporate global NTD elimination into newly proposed Sustainable Development Goals.

## Introduction: Version 1.0

The conceptual framework of the neglected tropical diseases (NTDs) began to take shape in the first few years following the 2000 Millennium Development Goals (MDGs), which called for efforts to combat “other diseases” alongside HIV/AIDS and malaria (MDG 6). It was recognized that a group of 13 or more parasitic and tropical infections have a number of common features including their chronicity and ability to cause long-term disabilities, especially among the extreme poor living in low- and middle-income countries [1]–[5]. Moreover, their public health importance (measured in disability-adjusted life years [DALYs]) was determined to be roughly equivalent to HIV/AIDS and malaria [3]. Critical to this framework was the concept of integrating control of at least seven of these NTDs—three soil-transmitted helminth infections, schistosomiasis, lymphatic filariasis (LF), onchocerciasis, and trachoma—through a low-cost “rapid impact package” of essential medicines now mostly donated by the multinational pharmaceutical companies [1]–[5]. The approach was considered “pro-poor” because the NTDs trapped people in poverty through their impact on child development, worker productivity, and other factors [6]. Today, national programs of NTD control and elimination are underway in at least 20 developing countries, predominantly through financial support of the Governments of the United States and United Kingdom [7], [8], in addition to a private END (Ending Neglected Disease) Fund [9], while under separate auspices, focused elimination and eradication efforts are underway for guinea worm [10] and leprosy [11]. In 2010, the World Health Organization (WHO) launched its first report on NTDs and expanded the list of major NTDs to 17 [12].

The “NTD movement” was not launched in a vacuum. Instead, it was constructed on a foundation of several key global health policies and documents that were advanced in the first few years of the new Millennium, including the MDGs, the *Report on the Commission on Macroeconomics and Health* led by Jeffrey Sachs, and *Our Common Interest: Report of the Commission for Africa*, commissioned by British Prime Minister Tony Blair [13]. In parallel, key WHO meetings were held in Geneva and Berlin; the latter sponsored by the German Organisation for Technical Cooperation (GTZ) [13]. These initiatives played an essential role for establishing a framework to integrate NTD control and elimination efforts through mass drug administration (also known as “preventive chemotherapy”) and allied approaches [13].

Following this first NTD period (V.1.0)—when the United States and United Kingdom Governments first initiated, and later scaled up, support for NTDs—we may be entering a new phase that began after 2010. Over the past three to four years, several new key global health policies and documents began to emerge (See Table 1). Such activities may alter how we conceptually frame NTDs for policymakers and proceed on expanded, or even new, paths for disease elimination. Here, we briefly report on how these developments in the context of a shifting policy landscape could have a significant impact on global NTD control and elimination.

**Table 1:** Comparisons between strategies and policies related to the NTDs: 2005–06 vs. 2013–14.

Category	V.1.0: 2005–06	V.2.0: 2013–14
Diseases	Seven Major NTDs; 13 Overall	17 NTDs
Highest disease burden	Hookworm & other STHis^1^, Schistosomiasis, Lymphatic filariasis	Visceral leishmaniasis, SchistosomiasisHookworm, and other STHis^1^, Lymphatic filariasis, Food-borne trematode infections
Rapid impact packages	Publication of policy papers	Implementation in >20 countries
Potential targets for elimination/eradiation in the coming decade	Dracunculiasis, Leprosy	Dracunculiasis, Leprosy, Lymphatic filariasis, Onchocerciasis, Trachoma, Sleeping sickness, Visceral leishmaniasis, Canine rabies
Special at-risk populations	Indigenous	Indigenous; Girls and Women
Incorporation into PEPFAR & GFATM	None	Limited; One success story in Togo
Links with NCDs	None	Published studies illustrating how NTDs are stealth causes of cardiovascular disease, cancer, and chronic pulmonary disease, among other conditions
Major Geographic Targets	Sub-Saharan Africa	Sub-Saharan Africa+G20 Countries
Key Policy Documents and Frameworks	Millennium Development Goals, Commission on Macroeconomics and Health, and Commission for Africa	London Declaration for NTDs, WHA Resolution, UN Women, UN Resolution on Prevention and Control of NCDs, Post-2015 MDGs, and Sustainable Development Goals

1 STHis = Soil-transmitted helminth infections

## Global Burden of Disease 2010 Study: The High Disease Burden NTDs

Early efforts to quantify the global burden of the major NTDs in DALYs found that these conditions were roughly equivalent to the DALYs lost from any of the “big three” conditions (i.e., HIV/AIDS, tuberculosis, and malaria), led by hookworm and other intestinal helminth infections, schistosomiasis, and lymphatic filariasis [3]. In December 2012, the major results of the Global Burden of Disease 2010 (GBD 2010) Study were published, which calculated the disease burden for almost 300 diseases and injuries, including 17 NTDs [14]–[16]. The results confirmed previous studies indicating that (with a few exceptions) the NTDs are mostly low mortality (measured in years of life lost—YLLs), but high morbidity (measured in years lived with disability—YLDs) conditions [14], [15]. However, GBD 2010 also found that in addition to the high disease burden helminthic NTDs identified in 2006 [3], the major kinetoplastid protozoan infections led by visceral leishmaniasis (VL), as well as sleeping sickness and Chagas disease, were also highly ranked in terms of DALYs lost annually [16]. The food-borne trematode infections, especially paragonimiasis and liver fluke infection, were also found to be important, as were selected emerging viral infections such as dengue or rabies [16]. Specifically regarding dengue, there is follow-up information pointing to an emerging pandemic with the troubling finding that up to 390 million dengue cases may occur annually [17]. Thus, any control or elimination strategy that fully embraces the major NTDs would need to address these specific diseases. Indeed, since 2010, several investigators have put forward suggestions for the global or regional elimination of sleeping sickness, VL, and canine rabies (the principal source of human infection) [18]–[20].

## UN Women: NTDs in Girls and Women

In 2010, the United Nations General Assembly established UN Women as an initiative for gender equality and empowerment of women, operating through partnerships, civil societies, and the governments of member states [21]. These efforts paralleled efforts led by then-U.S. Secretary of State Hillary Clinton, which were documented in the First Quadrennial Diplomacy and Development Review [22], and included the hiring of Melanne Verveer as the first United States Ambassador for Global Women's Issues [23]. There has also been increasing recognition of the disproportionate impact of NTDs on the health of girls and women [24], especially as a result of female urogenital schistosomiasis [25], [26], possibly now the most common gynecologic condition among African women [27], in addition to hookworm infections and dengue in pregnancy [28], [29], and mother-to-child transmission of Chagas disease [30], among other conditions [31]. However, to date, very few organizations or initiatives committed to girls and women have understood or yet fully embraced the importance of the NTDs. Global advocacy efforts will need to better link NTDs to adverse global women's health and direct UN Women to these diseases.

## Links to HIV/AIDS and Malaria and the Togo Study

One of the very first papers to highlight the concept of NTDs simultaneously pointed out their potential and actual links to HIV/AIDS, tuberculosis, and malaria [3]. Since then, a body of evidence has confirmed the associations between female urogenital schistosomiasis and HIV/AIDS [25]–[27], hookworm infections and malaria [32], [33], and other common links between NTDs and the big three diseases [34]. Despite an ever-growing evidence base for rational incorporation of NTD control and elimination efforts into the Global Fund to Fight AIDS, Tuberculosis, and Malaria (GFATM) and the United States President's Emergency Plan for AIDS Relief (PEPFAR) [34], [35], there has been only sporadic inclusion of NTDs into these programs. An important exception and success story was the recent publication of near elimination of lymphatic filariasis in Togo through mass drug administration, which was supported through the GFATM [36]. NTDs V.2.0 will require substantive and better incorporation into GFATM and PEPFAR.

## Hidden Links to the Non-Communicable Diseases

An important conclusion of the GBD 2010 Study is the increasing disease burden over the last 20 years that results from the non-communicable diseases (NCDs), e.g., cardiovascular disease, cancer, chronic pulmonary disease, and diabetes, with a roughly commensurate decrease in the disease burden resulting from infectious or communicable diseases [14]. In anticipation of this finding, in 2011, at UN headquarters in New York, a high level summit on the NCDs adopted a resolution for the prevention and control of these conditions [37]. Mostly unappreciated is the stealth contribution of the NTDs to the NCD disease burden in developing countries [38]. The NTDs are chronic and debilitating conditions that in many respects more closely resemble NCDs than most infections. For instance Chagas disease and loiasis are important causes of human cardiovascular disease; liver fluke and schistosomiasis are leading causes of cancer; and paragonimiasis and toxocariasis are leading causes of chronic pulmonary disease [38]. However, with the exception of a 2011 conference held in Boston led by Partners in Health [39], the role of NTDs has not yet entered high-level policy discussions on the NCDs.

## NTDs in the G20: A Foreign Policy Framework for the Poor among the Wealthy

**Figure 1:**
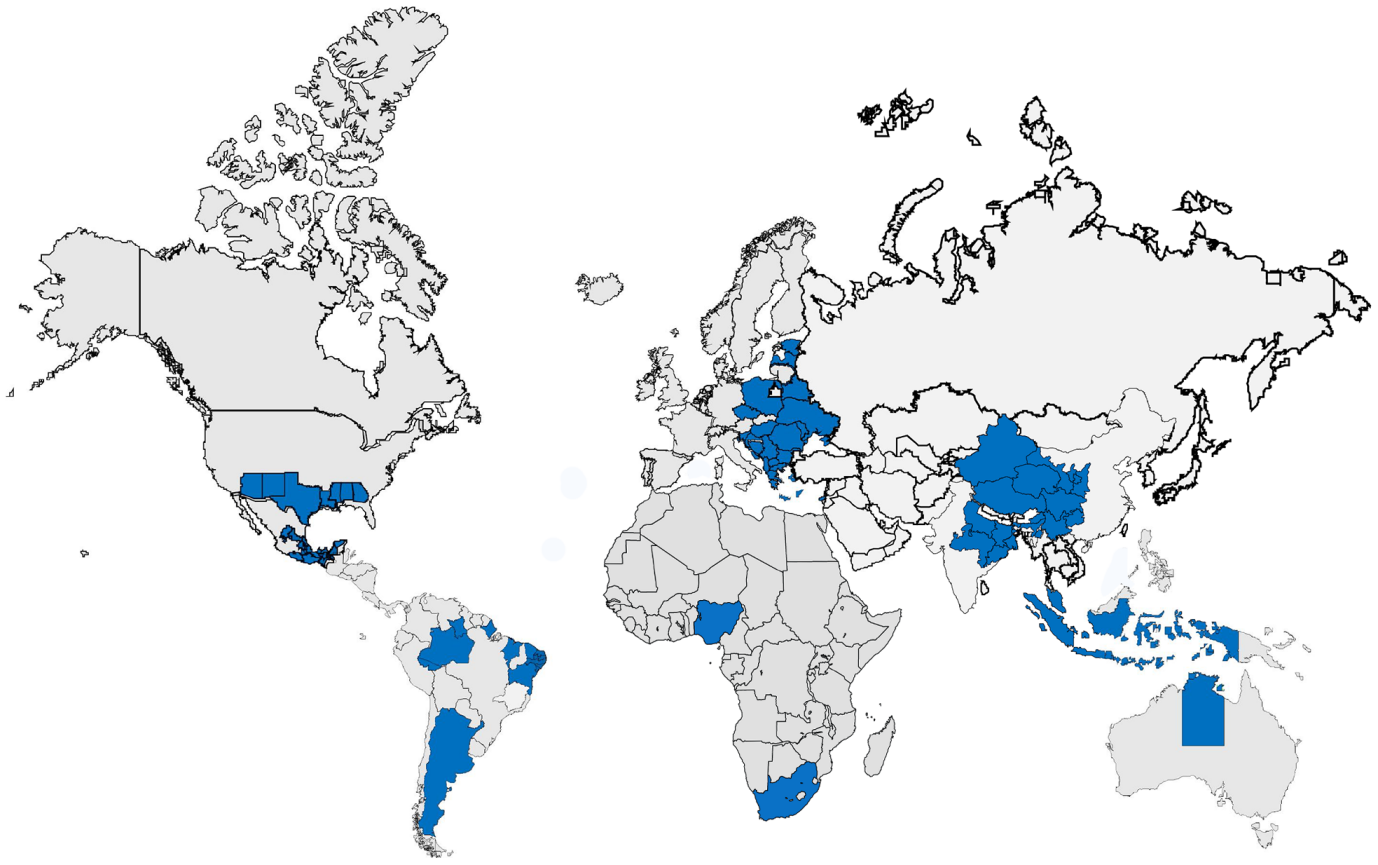
The poor living among the wealthy. Major areas of poverty in the G20 nations and Nigeria, where most of the world's NTDs occur (map prepared by Esther Inman). Figure originally appeared in “The Disease Next Door” by Peter Hotez, *Foreign Policy*, March 25, 2013, www.foreignpolicy.com. Information for Europe from Hotez PJ, Gurwith M. 2011. Europe's neglected infections of poverty. Int J Infect Dis 15: e611–9. Information for US and Mexico from Hotez PJ, Bottazzi ME, Dumonteil E, Valenzuela JG, et al. 2012. Texas and Mexico: sharing a legacy of poverty and neglected tropical diseases. PLOS Negl Trop Dis 6: e1497; Hotez PJ. 2012. Fighting neglected tropical diseases in the southern United States. BMJ 345: e6112; Hotez PJ. 2011. America's most distressed areas and their neglected infections: the United States Gulf Coast and the District of Columbia. PLOS Negl Trop Dis 5: e843; http://geo-mexico.com/?tag=economics&paged=2 accessed December 26, 2012; and http://www.plosntds.org/article/info%3Adoi%2F10.1371%2Fjournal.pntd.0000256, accessed December 26, 2012. Information for India from http://mapsofindia.com/maps/india/poverty.html, accessed December 25, 2012. Information for China from Hotez PJ. 2012. Engaging a rising China through neglected tropical diseases. PLOS Negl Trop Dis 6: e1599. Information for Brazil from http://web.worldbank.org/WBSITE/EXTERNAL/COUNTRIES/LACEXT/EXTLACREGTOPPOVANA/0,,contentMDK:22416581~pagePK:34004173~piPK:34003707~theSitePK:841175,00.html, accessed December 26, 2012; Information for Indonesia from http://sedac.ciesin.columbia.edu/data/set/povmap-small-area-estimates-poverty-inequality/maps/5, accessed December 26, 2012. Information for South Africa from http://www.cepf.net/where_we_work/regions/africa/maputaland/ecosystem_profile/Pages/socioeconomic_context.aspx, accessed December 26, 2012. Information for Australia from http://www.abs.gov.au/ausstats/abs.nsf/Latestproducts/1362.7Feature%20Article1Mar%202011?opendocument, accessed December 31, 2012. Information for Gran Chaco region of Argentina from http://en.wikipedia.org/wiki/File:GranChacoApproximate.jpg, accessed January 4, 2013.

**Figure 2:**
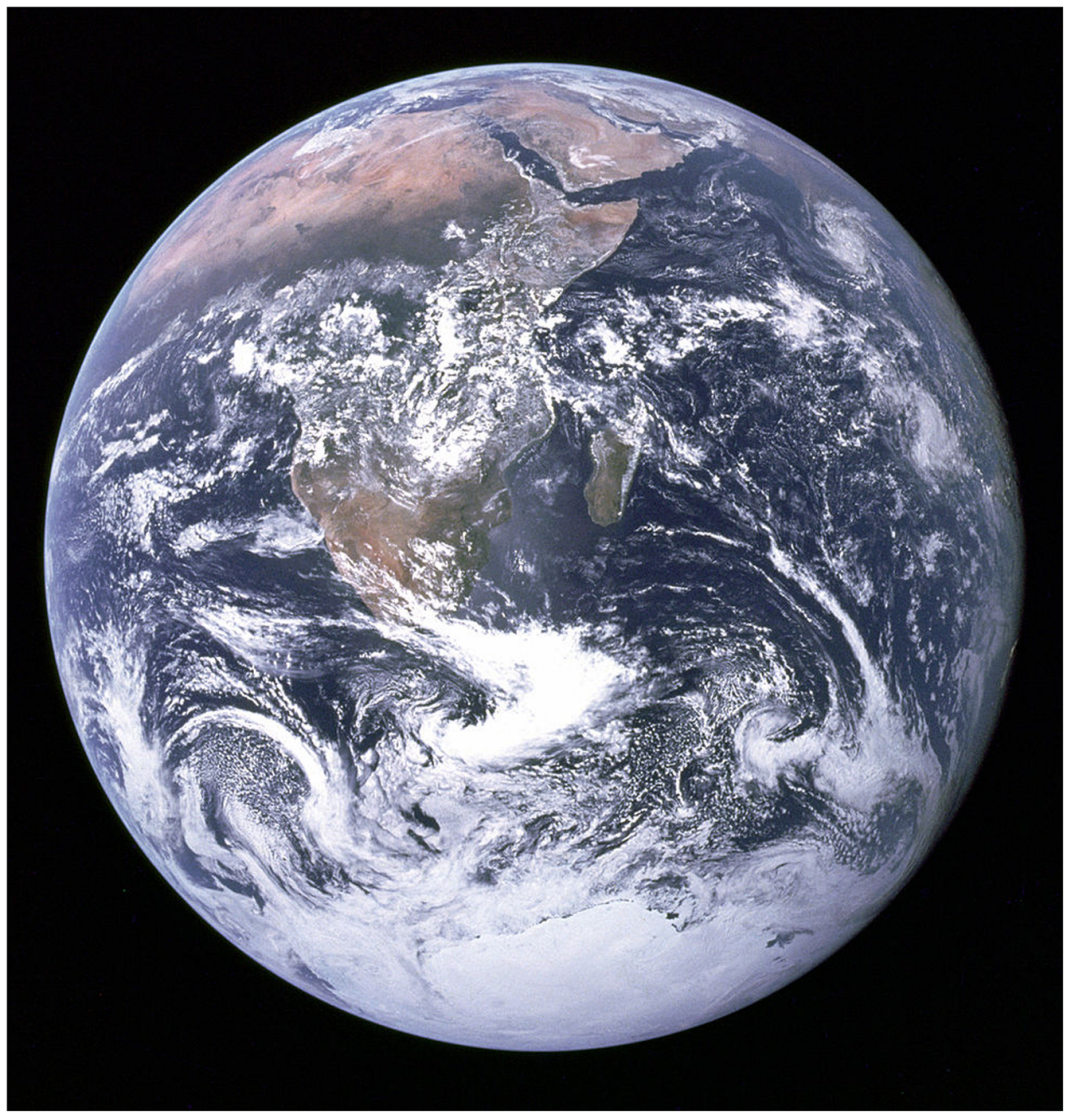
“The Blue Marble”—an international symbol of peace and healing—photographed by Astronauts, Eugene Cernan, Ronald Evans, and Jack Schmitt, December 7, 1972; http://visibleearth.nasa.gov/view.php?id=55418 **, accessed December 25, 2012.**

The rapid impact package was originally conceived in 2005 to emphasize mass drug administration efforts in Sub-Saharan Africa, where almost all of the world's cases of schistosomiasis and onchocerciasis were found, as well as more than one-third of the cases of trachoma and lymphatic filariasis [1]. However, in consideration of the high disease burden NTDs identified by the GBD 2010 Study, we will need to embrace additional NTDs, such as VL and food-borne trematode infections, among others. Somewhat paradoxically, we have found that many of these highest disease burden NTDs occur predominantly in the largest emerging market economies that comprise the group of 20 nations (G20), in addition to Nigeria, which also has one of the world's largest economies [40]. Table 2 shows a detailed analysis of the distribution of some of these diseases; a summary of which was published earlier in *Foreign Policy* [40]. Brazil, China, India, Indonesia, and Nigeria have the largest number of these NTDs, but even very wealthy nations, such as the United States, have a hidden burden of NTDs, especially in the American South [41]. Thus, while Sub-Saharan Africa remains a key region affected by NTDs, as we advance to NTDs V.2.0, we will need to embrace the observation that these conditions are also widespread among wealthier G20 countries. As shown in Figure 1, which was also published previously in *Foreign Policy*, it is the extreme poor living among the wealthy who disproportionately suffer from the world's NTDs [40]. This blurring between the health of developed and developing countries has been termed “blue marble health” [40], in reference to the photograph of Earth (and a symbol for peace) taken by the Apollo 17 astronauts [42], and is meant to foster a global dialogue on the importance of poverty as a key underlying factor for NTDs, regardless of where they occur (Figure 2). Blue marble health has the potential to be shaped into a new policy framework as it suggests that a substantial portion of the world's NTDs, up to one-half, or more, in some cases, may be eliminated if each of the G20 countries would assume greater responsibility for their own NTD problem and expand their indigenous control and elimination efforts. It was suggested previously that the new United States Department of State Office of Global Health Diplomacy (together with the UN and WHO) could have an important role in exerting diplomatic pressure on the G20 nations to aggressively pursue NTD disease control priorities [40].

**Table 2:** Neglected Tropical Diseases in the G-20 countries and Nigeria^1^.

Country	GDP Rank^2^	Cases of Visceral Leishmaniasis reported^3^	Cases of Schisto-somiasis^4^	Cases of Hookworm^5^^–^ 7	Population requiring MDA for lymphatic filariasis^8^^, ^ 9	Cases of Food-borne trematodiases^10^	Registered cases of leprosy^11^	Cases of Chagas disease^12^
United States	1							0.3 million
China	2	378	0.8 million	39 million		38 million	2,468	
Japan	3					<1 million		
Germany	4							
France	5	18						
Brazil	6	3,481	1.5 million	32 million	2 million		29,690	1.9 million
United Kingdom	7							
Italy	8	134						
Russia	9					<1 million		
India	10	34,918		71 million	610 million		83,187	
Canada	11							<0.1 million
Australia	12						7	
Mexico	14	7		<1 million			480	1.1 million
South Korea	15					1–2 million	265	
Indonesia	16		<0.1 million	62 million	123 million		23,169	
Turkey	18	29						
Saudi Arabia	20	34	<0.1 million	<1 million			5	
Argentina	27	8		2 million			723	1.6 million
South Africa	29		4.9 million	5 million				
Nigeria	40	1	28.8 million	38 million	106 million			
Total cases in G-20+Nigeria		39,008 reported	36 million	249 million	841 million	40 million	139,994	4.9 million
Total cases globally		58,227 reported	207 million	576 million	1.392 billion	56 million	181,941	8 million
% of cases in G-20+Nigeria		67%	17%	43%	60%	71%	77%	61%

1 A summary version of this table appeared in Reference 40.

2 https://www.cia.gov/library/publications/the-world-factbook/fields/2195.html, accessed December 31, 2012.

3 Alvar J, Velez IV, Bern C, Herrero M, Desjeux P, Cano J, Jannin J, den Boer M, the WHO Leishmaniasis Control Team. 2012. Leishmaniasis worldwide and global estimates of its incidence. PLOS One 7: e35671.

4 Steinmann P, Keiser J, Bos R, Tanner M, Utzinger J. 2006. Schistosomiasis and water resources development: systematic review, meta-analysis, and estimates of people at risk. Lancet Infect Dis 6: 411–25.

5 de Silva NR, Brooker S, Hotez PJ, Montresor A, Engels D, Savioli L. 2003. Soil-transmitted helminth infections: updating the global picture. Trends Parasitol 19: 547–551.

6 Bethony J, Brooker S, Albonico M, Geiger SM, Loukas A, Diemert D, Hotez PJ. 2006. Soil-transmitted helminth infections: ascariasis, trichuriasis, and hookworm. Lancet 367: 1521–32.

7 Li T, He S, Zhao H, Zhao G, Zhu X-Q. 2010. Major trends in human parasitic diseases in China. Trends Parasitol 26: 264–270.

8 http://www.who.int/neglected_diseases/preventive_chemotherapy/lf/db/index.html?units=minimal&region=all&country=all&countries=all&year=2011#, accessed December 24, 2012.

9 World Health Organization. 2012. Integrated preventive chemotherapy for neglected tropical diseases: estimation of the number of interventions required and delivered, 2009–2010. Weekly Epidemiol Rec 87: 17–28.

10 Furst T, Keiser J, Utzinger J. 2012. Global burden of human food-borne trematodiasis: a systematic review and meta-analysis. Lancet Infect Dis 12: 210–221.

11 World Health Organization 2012. Global leprosy situation, 2012. Weekly Epidemiological Record 87 317–28.

12 Bern C, Kjos S, Yabsley MJ, Montgomery SP. 2011. Trypanosoma cruzi and Chagas' disease in the United States. Clin Microbiol Rev 24: 655–81.

## The R&D Agenda and Vaccine Diplomacy

The importance of the G20 countries in effecting NTD control and elimination also extends to research and development (R&D). More than three-quarters of the world's public-sector global health R&D investments come from the Governments of the United States and United Kingdom, with so far minimal public investments from the other G20 nations [43]. New technologies for most of the highest disease burden NTDs, including new medicines, vaccines, diagnostics, and insecticides are urgently needed [44]. In 2010, a Consultative Expert Working Group (CEWG) of the WHO was established to advance a series of recommendations [45]. Among their findings, CEWG confirmed the inadequacy of funding for global health R&D funding, especially for product development, the lack of incentives for private-sector investment, and deficiencies in the current intellectual property system [45]. CEWG identified a need to explore innovative financing mechanisms that include financial commitments from countries, as well as a requirement to establish a global health R&D “observatory” under the auspices of the WHO to collect information and prioritize an R&D pipeline [45], [46]. In response, a US-based group of experts was convened to move forward recommendations on how to prioritize, coordinate, finance, and execute R&D to meet health needs in developing countries [46]. The U.S. group supported the concept of a global health observatory, and also supported expanding a financial base for public R&D expenditures through greater involvement from G20 countries other than the United States and United Kingdom, especially China, which has an active industrial investment portfolio in Sub-Saharan Africa [46]. In general, global health investments are not easy to encourage, given that most nations do not consider R&D as a component of overseas development assistance [46]. It was also noted that support would need to be provided for both public and private R&D institutions with a goal to promote true public-private partnerships [46]. More than a dozen developing country vaccine manufacturers were identified that currently participate in an important network alongside product development partnerships, midsize biotechs, and multinational pharmaceutical companies [46]. It was also noted that there is a new level of regulatory science required with a need for capacity building among many national regulatory authorities in low- and middle-income countries, as well as capacity building for research institutions in disease-endemic countries to partner with developing country manufacturers [46]. A revamped WHO Special Programme in Tropical Diseases (TDR) has also confirmed its commitment to capacity building. Finally, IP guidelines need to be revised to foster more open R&D efforts [46].

Another key aspect of the NTD R&D agenda is to promote scientific collaborations between institutions from countries regardless of their ideological perspectives, a concept sometimes referred to as “vaccine diplomacy” [47]. In the case of the United States, vaccine diplomacy is particularly relevant to some of the world's Islamic countries with technological sophistication, which even includes nuclear capabilities, such as Iran and Pakistan [47], [48].

## The London Declaration and a 2013 World Health Assembly Resolution

NTDs V.2.0 would target the high disease burden NTDs identified through GBD 2010 and take forward attempts at control and/or elimination for these and other diseases. Most of these activities are consistent with the goals and aspirations of an important 2012 London Declaration for NTDs for controlling or eliminating ten NTDs by the end of this decade [49]. The multinational pharmaceutical partner signatories of the London Declaration, as well as the WHO, World Bank, United States Government, and selected non-governmental organizations and donors (including the Gates Foundation), have agreed to supply the necessary drugs for mass drug administration and provide access to the rapid impact package to eliminate LF and blinding trachoma, and to control onchocerciasis, soil-transmitted helminth infections, and schistosomiasis. In NTDs V.2.0, the importance of these activities through policies established for girls and women by UN Women and the United States Ambassador for Global Women's Issues must be emphasized, while simultaneously working to incorporate NTD targets into GFATM and PEPFAR. The London Declaration also ensures the availability of drugs to eliminate sleeping sickness and control VL and Chagas disease [49]. NTDs V.2.0 would expand these activities to include vector control insecticidal spraying and making bednets available when appropriate. Finally the London Declaration would make available multi-drug therapies for leprosy elimination. NTDs V.2.0 must also emphasize high burden NTDs not mentioned in the London Declaration, including dengue, food-borne trematode infections, and rabies. A key feature of NTDs V.2.0 is working with 18 of the G20 governments not currently committed to supporting NTD control and elimination efforts, in addition to the Nigerian government, and to commit to these targets within their own national borders in addition to helping neighboring countries [40]. In the case of China there is added urgency to expand its outreach and technical capabilities to Sub-Saharan Africa [50]. For all nations, there is a need to consider these activities as a component of the neglected portion of their NCD commitments.

The London Declaration also renews a commitment from the major pharmaceutical partners and governmental and non-governmental organizations to promote R&D for “next-generation treatments and interventions.” As noted above, NTDs V.2.0 emphasizes the important role of public investments in R&D from all of the G20 countries, especially the major disease-endemic countries of Brazil, China, India, and Indonesia. Together, expanded use of available essential medicines for the NTDs and R&D represent the cornerstone of an audacious goal to eliminate all of the 17 NTDs [51]. NTDs V.2.0 recognizes the importance of London Declaration goals of access to “clean water, basic sanitation, improved living conditions, vector control, health education, and stronger health systems in endemic areas.”

In May 2013, the 66^th^ World Health Assembly approved a new resolution on the prevention, control, elimination, and eradication of NTDs. The resolution urges member states to take ownership of NTD programs, calling on international partners to provide “sufficient and predictable” funding, as well as emphasizing the importance of R&D for new technologies [52]. Several member states highlighted, in particular, the importance of a growing dengue fever pandemic.

## Concluding Statement: NTDs and the Sustainable Development Goals (SDGs)

The UN is working with governments, civil society, and key partners to advance post-2015 MDGs [53], and in 2012, hosted an important conference on sustainable development in Brazil, called Rio+20 (also known as Earth Summit 2012) to develop a new set of Sustainable Development Goals (SDGs) [54]. The SDGs would address important environmental issues not currently covered by the MDGs. In 2012, the UN Secretary-General announced the formation of a UN Sustainable Development Solutions Network (SDSN) that mobilizes scientific and technical expertise in support of the SDGs [55]. The NTDs are the most common infections in the world, thriving in the poorest communities, where they promote and cause poverty. They are the most common afflictions of girls and women and account for an important, but as yet unmeasured, component of the NCDs. The concept of blue marble health recognizes that NTDs are pervasive wherever poverty occurs, including not only the world's most devastated nations, but emerging market economies and wealthy countries in North America and Europe [40], [56]. It will be essential to incorporate NTDs and blue marble health into the new framework of the SDGs.
